# Solvent-Free Hydrogenation and Dehydrogenation of Quinoline and Quinaldine for the LOHC Concept

**DOI:** 10.1021/acsomega.6c00675

**Published:** 2026-02-19

**Authors:** Päivi T. Aakko-Saksa, Aino M. Mykkänen, Timo Repo

**Affiliations:** † University of Helsinki, A. I. Virtasen aukio, P. O. Box 50, 100014 Helsinki, Finland; ‡ 3259 VTT Technical Research Centre of Finland Ltd, P. O. Box 1000, 02044 VTT Espoo, Finland

## Abstract

As presented herein, *N*-heterocyclic quinoline (Q) and quinaldine (MeQ) represent promising liquid organic hydrogen carriers (LOHCs). They can be quantitatively hydrogenated to tetrahydro (TH) forms under mild reaction conditions (100 °C and 10 bar of H_2_) using Pt/C and Pd/C catalysts. It is noteworthy that the hydrogenation occurs without a solvent, yielding hydrogen storage capacities of up to 2.9 wt·%. Further hydrogenation of Q and MeQ to their decahydro (DH) forms requires the presence of a solvent. In addition to the hydrogenation of Q and MeQ, we succeeded in the dehydrogenation of the TH forms under solvent-free reaction conditions. This suggests that a pressure-controlled system with a single catalyst for hydrogenation and dehydrogenation of the MeQ/Q system could enable hydrogen storage under solvent-free reaction conditions. We also examined various substituted pyridine structures to comprehend the role of protective alkyl groups in hydrogenation. However, solvent-free quinaldine hydrogenation reactions are sensitive to steric changes surrounding pyridine nitrogen. Among the structural variations studied, MeQ with one methyl group has proven to be the most favorable.

## Graphical abstract

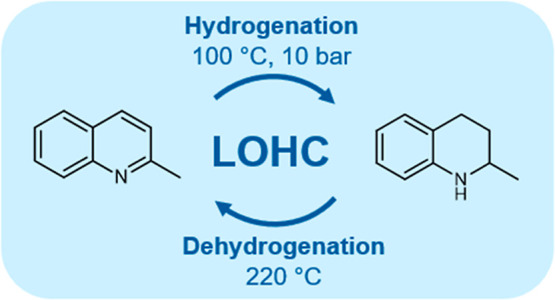

## Introduction

Hydrogen is a flexible energy carrier, potentially enabling the transition from fossil fuels to renewable energy. In addition to compressed and liquid forms of hydrogen, the efficient utilization of hydrogen requires different kinds of hydrogen storage, such as flexible “liquid organic hydrogen carriers” (LOHCs). The LOHC concept, which utilizes catalytic, reversible hydrogenation, and dehydrogenation of organic molecules, could be practical, effective, safe, robust, user-friendly, and flexible, depending on the chosen molecule.^1,2^ Dibenzyl toluene (DBT),^3^ benzyl toluene,^4^ and toluene^5,6^ are demonstrated as scalable LOHCs. These molecules have good thermal stability and relatively high hydrogen storage capacity (e.g., 6.2 wt·% for DBT-LOHC), but their hydrogenation and especially dehydrogenation require typically temperatures above 300 °C.^1,3,5–7^

Heterocyclic compounds with specific structures have also gained marked interest as LOHCs. Crabtree et al.^8^ identified favorable structural characteristics that make compounds suitable for the LOHC concept. These include bicyclic heteroarenes containing nitrogen atoms within a hydrocarbon ring, five-membered rings (excluding cyclopentane), and nitrogen atoms or substituents arranged at the 1,3-position (excluding imidazole). Additional favorable features are steric hindrance around the nitrogen atom and the presence of fused aromatic rings.

The structure of *N*-ethylcarbazole (NEC) follows many of these design features, and it has been widely studied as the LOHC compound. The hydrogenation of NEC to H12-NEC proceeds over a supported Ru or Pt catalyst at 150–170 °C and 50–70 bar H_2_;^3,8–13^ particularly, Ru/Al_2_O_3_ (5 wt·%) enables complete hydrogenation of NEC.^14–16^ However, the utility of NEC in the LOHC concept is limited as NEC itself is solid at room temperature.^1^ Other heterocyclic LOHC candidates are also being studied, such as 2-[(*n*-methylcyclohexyl)­methyl]­piperidine (H12-MBP)^17^ and perhydro-*N*-phenylcarbazole.^18^

When prioritizing the liquid form and low volatility of the LOHC components, we found quinaldine (2-methylquinoline, MeQ, bp 247.4 °C) and quinoline (Q, bp 237.1 °C) as promising candidates. Earlier study on toxicity of these LOHC candidates reported only slight toxicity toward soil bacteria.^19^ The selective hydrogenation of MeQ and Q is frequently performed to synthesize pharmaceutical active compounds and pesticides^20,21^ using homogeneous catalysts such as Ir, Ru, or Rh.^22,23^ Heterogeneous catalysts, such as Pd, Rh, Ru, Pt, Au, Ni, Co, or Cu, on various supports, including carbon, Al_2_O_3_, TiO_2_, SiO_2_, MgO, hydroxyapatite (HAP, Ca_10_(PO_4_)_6_(OH)_2_), and poly­(vinylpyridine), have also been studied. Pt, Pd, Rh, and Ru on carbon and alumina are recognized for their activity in hydrogenation reactions of *N*-heterocyclic arenes.^24–28^ Complete hydrogenation of MeQ or Q to their decahydro (DH) forms is reportedly achieved by using supported Rh and Ru catalysts in various solvents. A high yield of 99.3% for DH-Q was observed with the Rh/AlO­(OH) (aluminum oxyhydroxide) catalyst in *n*-hexane (125 °C, 8 bar, 3.5 h), whereas lower yields were noted when using a protic solvent.^26^ For example, Rh/Al_2_O_3_ gave moderate yields in *N*,*N*-diisopropylethylamine,^29^ hexafluoroisopropanol,^30^ and the pillared layered clay Rh-catalyst in isopropanol.^31^ In addition to Rh, Ru-based catalysts have also demonstrated the ability to hydrogenate MeQ and Q to their DH forms in cyclohexane. Ru/hydroxyapatite, which exhibits both acidic and basic characteristics, demonstrates outstanding performance (150 °C, 50 bar, 3 h)^32^ and high yield for DH-Q (>99%) was reported for the NanoRu@hectorite catalyst at 100 °C, 60 bar, over 3 h.^33^ From this background, Ru nanoparticles supported on glucose-derived carbon spheres (Ru/CSP) are an exceptional example, as they yielded 95.2% DH-Q in water (120 °C, 20 bar, 4 h).^34^

In the studies presented above, tetrahydro (TH) forms of Q and MeQ often appeared alongside the DH forms. In fact, TH forms can be selectively prepared under mild reaction conditions by selecting an appropriate solvent and catalyst.^29^ For instance, Pd/MgO exhibits high activity for the (Py)­TH-Q synthesis,^35^ as do Pd/NCNs-N_*x*_ (4 bar, 40 °C) and Ru/N-doped carbon (Ru 7 nm, 30–50 °C, 10 bar). Also, Pd nanoparticles (4 nm) on tannin grafted onto collagen fiber were reportedly active for the hydrogenation of Q to py-THQ in [bmim]­[BF4] (bmim = 1-butyl-3-methylimidazolium cation, 60 °C, 20 bar, 1 h.^36^ Generally, challenges in the hydrogenation of MeQ or Q are considered to be due to the potential dative bonding of Lewis basic nitrogen on catalytically active metal surfaces, which likely causes thermodynamically favorable intermediates.^37^ This interaction also guides the hydrogenation reaction, rendering it remarkably selective toward the pyridine ring while avoiding the reduction of the fused benzene rings. Reportedly, py-TH-Q is preferred in polar,^38^ neutral, or weakly acidic media,^26^ while hydrogenation to bz-TH-Q (or to DH-Q) has been achieved mainly with nonpolar solvents (see above).^26^

MeQ and Q are liquids, and intriguingly, they also possess a high theoretical hydrogen storage capacity (DH-Q = 7.2 wt·% and DH-MeQ = 6.6 wt·%). From the studies above, it can be concluded that the solvents’ usage has proven necessary to attain both DH-Q and DH-MeQ. However, the necessity of using a solvent will inevitably diminish their hydrogen storage capacity in the LOHC concept, increase the energy demand for heating and generate waste, while solvent-free scope offers a sustainable and efficient approach for hydrogen storage. While Rh/AlO­(OH) is capable of catalytic hydrogenation of Q in the absence of a solvent over an extended reaction time of 30 h,^27,39^ practical solvent-free hydrogenation and dehydrogenation reactions of Q and MeQ have been lacking. Herein, a series of heterogeneous Pt, Pd, Rh, and Ru catalysts were studied for the solvent-free hydrogenation of quinoline and quinaldine. Quantitative TH-Q and TH-MeQ yields without solvent were recorded (Scheme 1). Alongside hydrogenation, dehydrogenation of hydrogenated products, which is central to the LOHC concept, was achieved successfully under mild conditions. The TH-Q cycle, therefore, offers a moderate hydrogen storage capacity of up to 2.9 wt·%, which could be feasible in applications where mild reaction conditions are essential, such as capturing hydrogen leakages that would otherwise contribute to global warming.^40^ To explore the challenges of Q and MeQ hydrogenation, we also examined 2,6-lutidine as a symmetric, methyl-protected pyridine along with 2,6-dimethyl-1,8-naphthyridine and the well-known *N*-ethylcarbazole (NEC) for comparison.

**Scheme 1:**
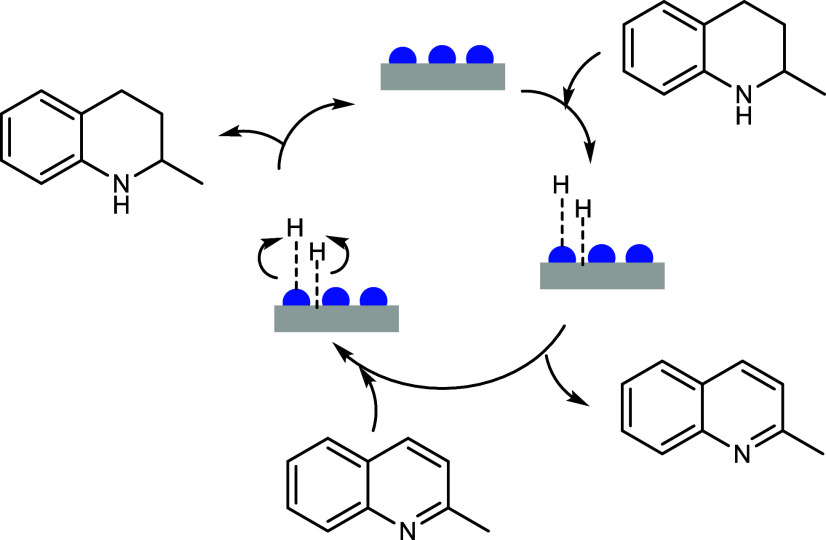
Catalytic Cycle for Hydrogenation of Quinaldine (MeQ) to 1,2,3,4-Tetrahydroquinaldine (py-TH-MeQ) and its Dehydrogenation to MeQ

## Experimental Section

### Materials

Quinaldine (CAS 91-63-4), quinoline (CAS 91-22-5), 1-methyl naphthalene (CAS 90-12-0), lutidine (CAS 108-48-5), and *N*-ethyl carbazole (CAS 86-28-2, >97%) were purchased from Sigma-Aldrich, and dibenzyltoluene (CAS 26898-17-9) was provided by Sasol GmbH. The hydrogenated TH-MeQ and TH-Q used in the dehydrogenation experiments were prepared in-house.

Catalysts used in the hydrogenation experiments were Pt/C 5 wt·%, Pd/C 5 wt·%, Rh/AlO­(OH) 5 wt·%, and Ru/Al_2_O_3_ 5 wt·%. All catalysts were from Sigma-Aldrich. Properties reported by others for similar catalysts: Pt/C 5 wt·%: Pt dispersion 49.4%, specific surface area (SSA) 1608 m^2^·g^–1^, pore volume (PV) 0.98 cm^3^·g^–1^, pore size (PS) 4.7 nm.^24^ Pd/C 5 wt·%: Pd size 2.4 nm, dispersion 22–36%, SSA 1120 m^2^·g^–1^; grain size 23 mm.^25^ Rh/AlO­(OH) 5 wt·%, Rh size 3.2 nm;^26^ dispersion 28.7%, SSA 616 m^2^·g^–1^, PV 0.85 cm^3^·g^–1^, PS 2.9 nm^27^ Ru/γ-Al_2_O_3_ 3 wt·%, SSA 150 m^2^·g^–1^.^28^

### Hydrogenation Experiments

Hydrogenations were conducted in a Roth autoclave in a 300 mL glass insert placed in the reactor. Aluminum foil placed on top of the insert aimed to prevent the evaporation of substrate from the insert. The autoclave was flushed three times with nitrogen (AGA, 99.95%), 10 bar, before introducing hydrogen (AGA, 99.9%). After the reaction, the autoclave cooled for 30–45 min before hydrogen was released. Samples were transferred to vials. In reactions, 4 mL of liquid substrates (in mixing experiments 2 mL + 2 mL) and 0.25 mol·% catalyst were placed in the insert, while for solid substrates, the respective amount was weighed. Temperatures were between 80 and 170 °C, reaction times were 1–6 h, and hydrogen pressures were 5–50 bar. The stirring speed was 990 rpm.

### Determination of Hydrogenation Degree

Gas chromatography (GC) with a flame ionization detector (FID) and mass spectrometer (MS) were used to determine the hydrogenation degree. Two instrument setups were used. The first setup consisted of GC Agilent 6890N (column HP-INNOWAX) and MS Agilent 5973 (column Agilent J&WDB-1MS). Another setup included Agilent 5977B GC/MSD with columns HP-5MS 5% Phenyl Methyl Silox and HP-5MS Ultra Inert. Initial temperature was 50 °C, ramp rate was 25 °C/min, and final temperature was 280 °C. The catalyst was removed from samples by HPLC filtration, silica, or both before analyses. Samples were diluted with ethyl acetate, 1.0 mg/mL for the first setup and 0.1 mg/mL for the second setup, and an internal standard (ISTD), acetophenone, was added. The calibration curve was determined using standard solutions containing ISTD (1.054 mg/mL) and quinaldine (1.96 mg/mL, 0.98 mg/mL, and 0.49 mg/mL) in ethyl acetate. Ratios of GC-FID peak areas for quinaldine and acetophenone were used to determine the amount of TH-MeQ in samples.

### Dehydrogenation Experiments

A mixture of substrate and catalyst was heated in a round-bottom flask while being stirred with a magnetic stirrer (uniSTIRRER 7, LLG) at 300 min^–1^. The molar ratio of the substrate and active metal of the catalyst was targeted at 400:1 (0.25 mol·%), and molar ratios based on weight were used in calculations.

### Determination of the Dehydrogenation Degree

In the dehydrogenation experiments, NMR analyses with a Varian 300 MHz were conducted to verify the degree of dehydrogenation. Number of scans was 32. The catalyst was removed from the samples by HPLC filtration. The filtrate was diluted with dimethyl sulfoxide-d6 (DMSO-*d*_6_) prior to the NMR analyses. Additionally, formed hydrogen was collected in the measuring glass, and readings were recorded periodically to obtain a time series of released hydrogen.

## Results and Discussion

### Hydrogenation of Quinaldine and Quinoline without Solvents

In this study, we primarily focus on the solvent-free hydrogenation of MeQ and Q with commercial catalysts, Pt/C, Pd/C, Rh/AlO­(OH), and Ru/Al_2_O_3_, which have high specific surface areas (150–1608 m^2^·g^–1^) and small sizes of active metals (Pd 2.4 nm 25 and Rh 3.2 nm).^24–28^ The reactions studied covered complete hydrogenation of MeQ or Q, which would lead to their decahydro forms, DH-MeQ and DH-Q, while tetrahydro intermediates are py-TH-MeQ, py-TH-Q, bz-TH-MeQ, and bz-TH-Q (Schemes 2 and 3). For the LOHC concept, the selectivity in synthesizing TH forms, such as the hydrogenation of either pyridine or benzene rings, is unnecessary, as both structures exhibit a similar hydrogen storage capacity.

**Scheme 2:**
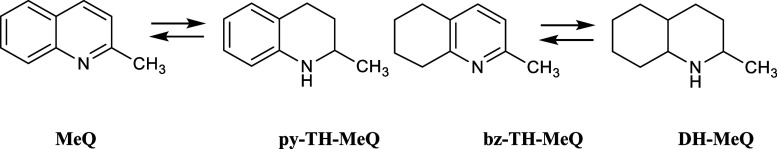
Methylquinaldine MeQ and its Partially Hydrogenated Intermediates py-TH-MeQ and bz-TH-MeQ as Well as the Fully Hydrogenated Decahydroquinaldine DH-MeQ

**Scheme 3:**
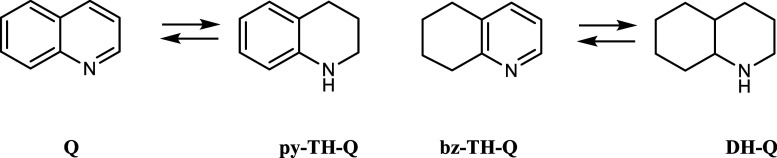
Quinoline (Q), Its Partially Hydrogenated Intermediates py-TH-Q and bz-TH-Q, and Decahydroquinoline (DH-Q)

The partial hydrogenation of MeQ and Q proceeded effectively in the absence of solvents under mild reaction conditions (Table 1). In the series, the Pt/C catalyst was particularly active in the hydrogenation reaction, achieving quantitative conversion of MeQ to its TH-MeQ form at a pressure of 10 bar H_2_ and temperatures ranging from 80 to 100 °C over a period of 2 h. The yield of TH-Q under these conditions was 93% (Figure 1a–d). Furthermore, a TH-MeQ yield of over 95% was sustained even when the pressure was lowered to just 5 bar H_2_ at 150 °C within a 1 h reaction time.

**Table 1:** Solvent-Free Hydrogenation of MeQ and Q into Their TH and DH Forms.^b^

catalyst^a^	substrate	pressure (bar)	temperature (°C)	time (h)	TH forms (%)	DH forms (%)
Pt/C	MeQ	40	150	2	100	0
Pt/C	MeQ	30	150	2	100	0
Pt/C	MeQ	20	150	2	100	0
Pt/C	MeQ	10	150	2	100	0
Pt/C	MeQ	5	150	2	98	0
Pt/C	MeQ	33	130	2	90	0
Pt/C	MeQ	10	80	2	100	0
Pt/C	MeQ	10	100	1	95	0
Pt/C	Q	40	150	2	100	0
Pt/C	Q	21	150	2	100	0
Pt/C	Q	10	150	2	100	0
Pt/C	Q	10	100	2	93	0
Pd/C	MeQ	40	150	2	100	0
Pd/C	MeQ	20	150	2	100	0
Pd/C	MeQ	10	100	2	86	0
Pd/C	Q	40	150	2	100	0
Ru/A	MeQ	40	150	2	39	0
Ru/A	MeQ	30	150	2	30	0
Ru/A	MeQ	20	150	2	27	0
Ru/A	Q	40	150	2	40	0

a A = Al_2_O_3_.

b The amount of catalyst is 0.25 mol·%. The molar ratio of the substrate to the active metal of the catalyst was 400:1.

**Figure 1:**
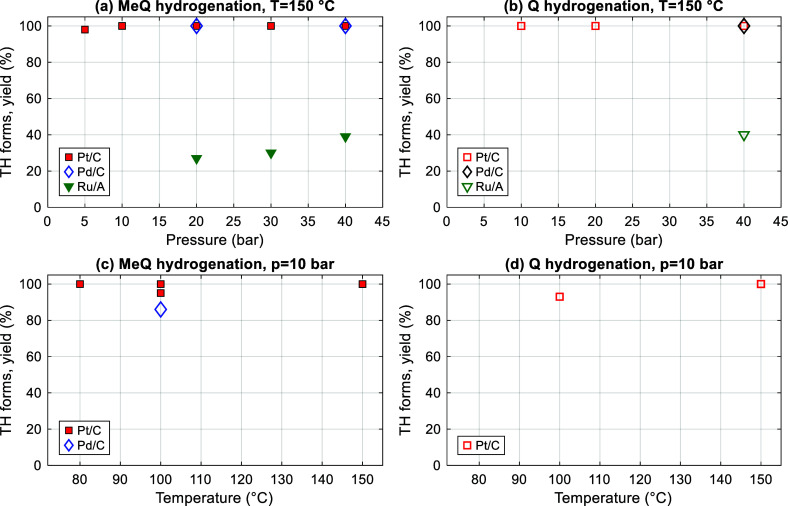
Solvent-free hydrogenation of (a) MeQ and (b) Q at a temperature of 150 °C and varying pressures and (c) MeQ and (d) Q at a hydrogen pressure of 10 bar and varying temperatures. The molar ratio of the substrate and active metal of the catalyst was 400:1.

With Pd/C, quantitative hydrogenation of Q and MeQ was achieved at a pressure of 40 bar and a temperature of 150 °C in 2 h. At 100 °C and a pressure of 10 bar of H_2_, under the conditions where Pt/C achieved a quantitative yield of TH-MeQ, the Pd/C catalyst hydrogenated MeQ with a yield of 86% (Figure 1b). In all these cases, the hydrogenation was selective for the pyridine ring of Q and MeQ (see Figure S1 in Supporting Information), and the yields of TH-MeQ were slightly higher than those of TH-Q.

Among the series of catalysts, Ru/Al_2_O_3_ exhibited the lowest activity, which may be attributable to its lower specific surface area compared with the other catalysts. It resulted in only 27–39% conversion of MeQ and Q into their corresponding TH forms at 150 °C and 20–40 bar over 2 h reaction time (Table 1, Figure 1a). Since the Ru/Al_2_O_3_ catalyst is active in the hydrogenation of DBT, 1-methylnaphthalene (MeN), and NEC, we studied the hydrogenation of the mixtures of MeQ, Q, DBT, MeN, and NEC (Table S1 in Supporting Information). With the Ru/Al_2_O_3_ catalyst, DBT improved the conversion of MeQ to TH-MeQ, while MeN did not substantially affect the conversion and NEC inhibited the hydrogenation of MeQ.

### Hydrogenation of Quinaldine and Quinoline with Solvents

In our experiments, the hydrogenation of MeQ over the Rh/AlO­(OH) catalyst proceeded to DH-MeQ only when iPrOH was used as the solvent (Table 2, Figure S2 in Supporting Information). The quantitative reaction was obtained at 40 bar of H_2_ and 100 °C for an extended reaction time of 6 h. If the hydrogen pressure was reduced to 20 bar, the hydrogenation reaction remained incomplete; the yield of DH-MeQ was 33%, and the yield of TH was 66%. Although high yields of DH-Q have been previously reported for the hydrogenation of Q over the Rh/AlO­(OH) catalyst also in nonpolar solvents such as *n*-hexane,^26^ the use of alternative solvents here resulted in only TH forms (Table 2).

**Table 2:** Hydrogenation of MeQ to the TH and DH Forms in i-Propanol (iPrOH) and *n*-Hexane (nH).^b^

catalyst, solvent^a^	pressure (bar)	temperature (°C)	time (h)	H4 form (%)	H12 form (%)
Rh: MeQ + nH	8	100	3	57	0
Rh: MeQ + nH	15	100	3	63	0
Rh: MeQ + nH	25	100	3	65	0
Rh: MeQ + nH	40	100	6	98	0
Rh: MeQ + iPrOH	40	100	6	0	100
Rh: MeQ + iPrOH	20	100	6	66	33
Pt/C: MeQ + iPrOH	40	100	6	100	0

a Rh = Rh/AlO­(OH).

b The amount of catalyst is 0.25 mol·%.

It is possible that iPrOH may also function as a transient hydrogen donor in this reaction, facilitating a heterolytic mechanistic pathway for the hydrogenation of pyridine. iPrOH is a known hydrogen donor solvent.^41^ With the Pt/C catalyst, the hydrogenation reactions yielded exclusively TH-MeQ when the hydrogenation reaction was conducted in iPrOH (Table 2). To summarize the results above, complete hydrogenation of MeQ to its DH-form can be achieved using a solvent; however, solvents are detrimental to a LOHC’s hydrogen storage capacity. In addition to reducing the hydrogen storage density, solvents introduce several other negative effects, such as increased energy demand for heating and losses due to evaporation, as well as lower hydrogen purity.

### Hydrogenation of *N*-Heterocycles with Modified Structures

We also aimed to explore the potential of modifying the pyridyl structures by introducing protecting groups to reduce the catalyst surface adsorption of pyridyls and TH intermediates formed during the hydrogenation of MeQ and Q. To study these structural aspects, we extended the study to 2,6-dimethyl-1,8-naphthyridine (DMeN, Scheme 4), 2,6-lutidine (Scheme 5), and NEC (Scheme 6). DMeN comprises two merged pyridine rings, where the two methyl groups provide different steric environments for the pyridine nitrogens. In the lutidine, two methyl groups could induce steric hindrance around the N atom, affecting the dative bonding between the catalyst’s active sites. Nevertheless, they are not suitable LOHC candidates due to the solid state of DMeN and the low boiling point of lutidine from the application’s perspective (144 °C).

**Scheme 4:**
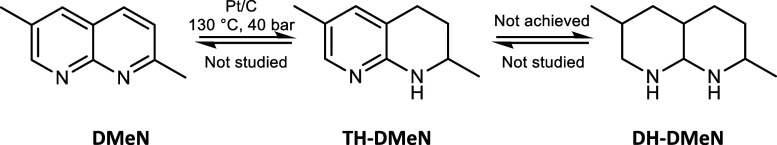
A LOHC System Consisting of 2,6-Dimethyl-1,8-naphthyridine (DMeN) and its Hydrogenated Forms

**Scheme 5:**
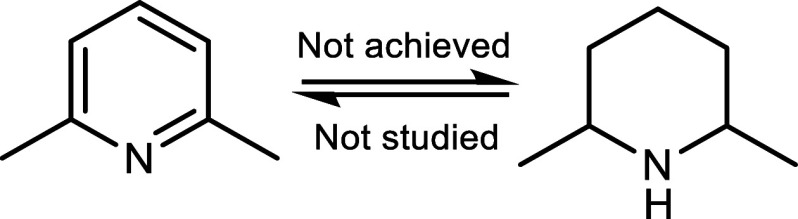
A LOHC System Consisting of 2,6-Lutidine and 2,6-Dimethylpiperidine

**Scheme 6:**
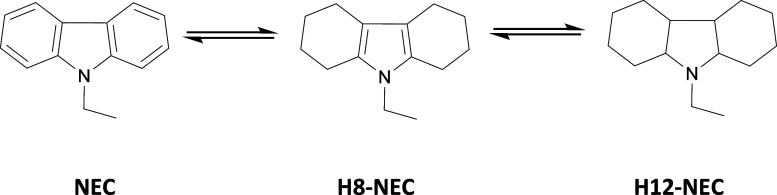
A System Consisting of NEC, H8-NEC, and H12-NEC

The hydrogenation of DMeN with a Pt/C­(1 wt·%) catalyst at 130 °C and 40 bar for 3 h resulted in the hydrogenation of one of the two pyridyl rings. The hydrogenation of DMeN led to the hydrogenation of the ring containing the methyl group on the carbon adjacent to the *N*-atom, while the other ring, which had a methyl group at the 6-position, remained unchanged (NMR, Figure S3 in Supporting Information). The methyl group at position 2 is evidently sterically favorable and likely aids in the desorption of the pyridine ring or its hydrogenated form, piperidine, from the active catalyst surface. DMeN and its partially hydrogenated products were solid. 2,6-Lutidine, which features two methyl groups adjacent to *N*-atom, showed no signs of hydrogenation under the mild, solvent-free reaction conditions (100 °C, 10 bar, 2 h, 0.25 mol·% of Pt/C (5 wt·%)). Most likely, the two methyl groups adjacent to the *N*-atom sterically shield the pyridine nitrogen, hindering the adsorption of pyridine onto the catalyst and thereby preventing hydrogenation under these reaction conditions. It can therefore be stated that solvent-free quinaldine hydrogenation reactions are sensitive to steric changes around the pyridine nitrogen, and of the structural variations now studied, MeQ is the most favorable. However, regardless of the methyl substitution, the hydrogenation selectivity of Q and MeQ remains the same, and there is no indication that the steric hindrance posed by the methyl group adjacent to the *N*-atom alleviates the hydrogenation of the benzo ring under the same reaction conditions.

The hydrogenation of NEC to octahydro-*N*-ethylcarbazole (H8-NEC) and dodecahydro-*N*-ethylcarbazole (H12-NEC) as well as the dehydrogenation of hydrogenated forms are known reactions (Scheme 6).^14–16^ Structurally, in NEC, the 5-membered heterocycle includes a tertiary nitrogen fused with two aromatic rings. Moreover, the ethyl group, acting as a protective substituent on the *N*-atom, increases steric hindrance and reduces the adsorption of the *N*-atom on the catalytically active sites. This is essential for easy hydrogenation, and unlike in Q and MeQ, hydrogenation of NEC starts from the outer phenyl groups, where less steric hindrance appears.^15,16^ ^,^ ^15,42,43^ The solid form of NEC at normal temperature and the dealkylation of nitrogen at elevated temperatures during dehydrogenation are significant drawbacks of its otherwise beneficial structural features, which may limit NEC’s use in certain LOHC applications.^10^

In our solvent-free experiments, Ru/Al_2_O_3_ (5 wt·%, 400:1 molar ratio) achieved full conversion of NEC into hydrogenated products, with selectivities of 28–55% to H12-NEC and 39–63% to H8-NEC at 150–170 °C, 50 bar, over 3–6 h (Table 3). The product mixture represented approximately 4.8 wt·% hydrogen storage capacity. Interestingly, a higher yield was attained at 150 °C compared to 170 °C when the reaction time was extended, which may be due to the reversibility of the reaction at high temperatures and selectivity toward H8-NEC and H12-NEC forms, as no byproducts were detected. We achieved almost complete solvent-free hydrogenation of NEC to H12-NEC using a Ru on alumina catalyst with Pt- or Rh-based promoters under relatively mild reaction conditions (170 °C, 50 bar). The activity of the Pd/C promotor was low, despite Pd’s capability to promote hydrogen spillover.^44^ Other favorable characteristics of Pd include a low tendency for deactivation^45^ and the ability to form alloys with other metals.^46^ Neither Pt/C nor Pd/C alone significantly hydrogenated NEC.

**Table 3:** Hydrogenation of NEC to its H8 and H12 Forms by Using Ru-, Pt-, Pd-, and Rh-Based Catalyst Systems

catalyst^a^	pressure (bar)	temperature (°C)	time (h)	H8 (%)	H12 (%)
Ru/A + Pt/C	50	170	3	0 (0^b^)	100 (0^b^)
Ru/A + Pd/C	50	170	3	52 (4^c^)	48 (0^c^)
Ru/A + Rh/AlO(OH)	50	170	2;5	84;3 (100^d^)	16;97 (0^d^)
Ru/A	50	170	6;2	63;99 (39; 45^e^)	35;1(28; 55^e^)

a A = Al_2_O_3_.

b Pt/C only.

c Pd/C only.

d Rh/AlO­(OH) only, 40 bar, 150 °C, 3h.

e 50;45 bar, 150 °C, 3h.

### Dehydrogenation of TH-MeQ

The dehydrogenation of TH-MeQ and TH-Q is essential to the LOHC concept. In our experiments, TH-MeQ was successfully dehydrogenated using a Pt/C catalyst, achieving a quantitative yield in 4.5 h when the temperature was raised to 220 °C. Based on the GC–MS and ^1^H NMR spectra, no TH form remained (Figure 2). With the same catalyst, the dehydrogenation of TH-Q occurred in 3.3 h as the temperature was elevated in 10 °C increments from 137 to 273 °C. Volumetric measurements of the released hydrogen indicated a release of 73% of the theoretical maximum. According to the ^1^H NMR spectrum, the TH form was still present. Although the literature indicates the successful hydrogenation of Q to THQ, and its subsequent dehydrogenation on a Pt nanowire, this was earlier accomplished using a solvent scope.^47^

**Figure 2:**
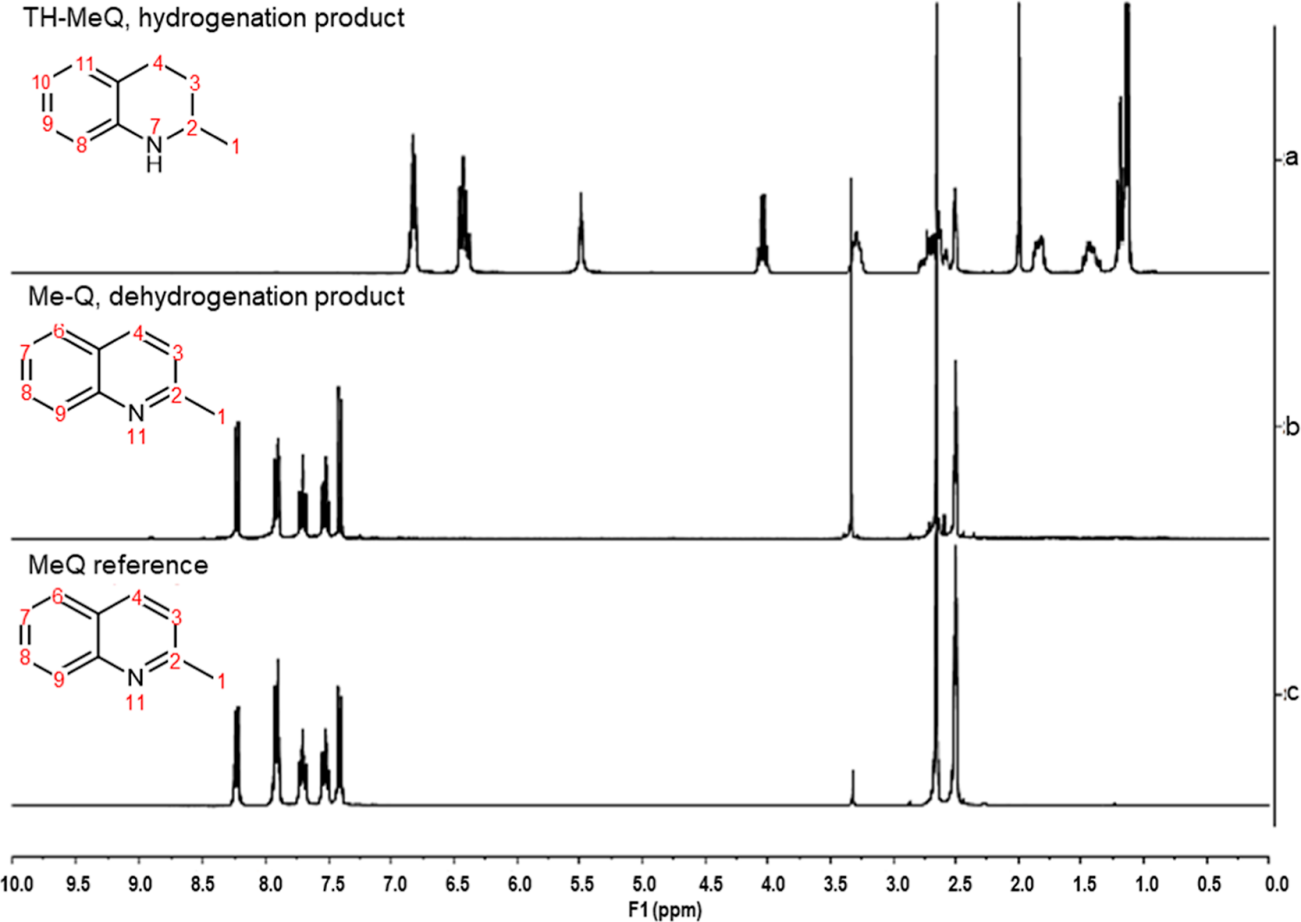
^1^H NMR spectra (in DMSO) for the (a) TH-MeQ product used in dehydrogenation, (b) dehydrogenated product, and (c) quinaldine (MeQ) reference spectra. Assignment of shifts in ^1^H NMR spectra is presented in Table S2.

Utilizing the same dehydrogenation setup, we quantitatively dehydrogenated H12-NEC with a Pt/C catalyst at 220 °C for a duration of 13 min. The dehydrogenation of H12-NEC is a well-known reaction, reportedly proceeding initially through the activation of C–H bonds within the 5-membered ring of H12-NEC, leading to the formation of H8-NEC before the dehydrogenation of the 6-membered rings.^9^

### Challenges in Hydrogenation of Quinaldine and Quinoline

The complete hydrogenation of MeQ and Q without solvents has remained elusive in both the literature and our investigation. Accordingly, solvents’ role in selectivity and complete hydrogenation of MeQ and Q is irreplaceable in this reaction.

The main challenge preventing complete hydrogenation of MeQ and Q is the dative bonding between the free electron pair of the pyridine ring and the catalyst’s active site, which hinders the adsorption and hydrogenation of the benzene ring. Furthermore, desorption of the pyridine structure requires a higher temperature than that of the benzene ring.^21^ In the continuation, tailored catalysts with basic or acidic nature could be interesting based on studies on the role of catalyst supports to reduce the adsorption of reaction intermediates on the catalyst active sites.^31,33,35,38,48,49^ For example, basic support (Pt/MgO) is reported to involve a strong hydrogen bonding between the *N*-atom and a basic site on the support, enabling hydrogenation of the benzene ring.^49^ *N*-doped supports^21^ and the introduction of OH-groups in catalyst are also interesting options.^21,36,50,51^ Acidic supports may promote undesired side reactions alongside their beneficial characteristics, such as resistance to poisoning.^45,52^ To achieve the maximum hydrogen storage capacity, complete hydrogenation of Q and MeQ would be required. In this case, a small amount of solvent, as well as tailoring of the catalyst and consideration of protection groups in the substrate, would warrant further attention.

## Conclusions

MeQ and Q are intriguing LOHC candidates with high theoretical hydrogen storage capacities, 6.6 and 7.2 wt·%, respectively. However, the use of solvents is crucial for the hydrogenation of MeQ and Q to their fully saturated decahydro forms, which significantly diminishes the hydrogen storage capacity of the LOHC concept. Selective hydrogenations of pyridine or benzene rings in MeQ and Q have also been reported in the presence of solvents. It has been proposed that solvents are essential for the complete hydrogenation of MeQ and Q, as they reduce the adsorption of TH-intermediates onto the active sites of the catalyst. To date, the hydrogenation of MeQ and Q has primarily been examined in the presence of various solvents. In contrast, our study focuses on the solvent-free hydrogenation of MeQ and Q and explores their potential as LOHC components. We also included analogous reactions in solvents for comparison. For example, quantitative hydrogenation of MeQ to DH-MeQ was only achieved when using isopropanol as a solvent and Rh/AlO­(OH) as a catalyst under elevated pressure (40 bar) and temperature (100 °C) for 6 h. We also examined the hydrogenation challenges associated with the structure of MeQ and Q, particularly the function of the dative bonding between the pyridyl and the metallic nanoparticles; however, the varied methyl substitution pattern did not show improved hydrogenation efficiency.

As shown here, Q and MeQ can be quantitatively hydrogenated to TH-Q and TH-MeQ in a solvent-free environment under mild reaction conditions (100 °C, 10 bar) and in a relatively short reaction time of 1–2 h using Pt/C and Pd/C catalysts. Remarkably, the reaction is reversible, and the dehydrogenation of the hydrogenated TH form of MeQ was quantitative at 220 °C (Scheme 7). In comparison, the dehydrogenation of the TH form of Q was partial, even at 270 °C. To the best of our knowledge, the solvent-free hydrogenation and dehydrogenation of Q and MeQ is unprecedented. This enables a practical MeQ-based concept for hydrogen storage with a capacity of up to 2.9 wt·%. Such a system could potentially be feasible for applications where mild reaction conditions are essential, such as capturing hydrogen leakages that would otherwise contribute to global warming.^40^ The ability to adjust the hydrogenation and dehydrogenation modes by altering the pressure is a significant advantage that aids in the development of a streamlined, pressure-controlled LOHC system using a single catalyst.

**Scheme 7:**
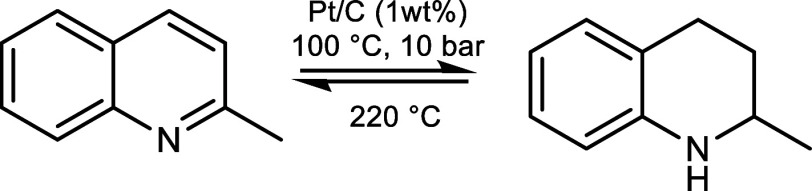
LOHC Pair Is Based on MeQ and Its Hydrogenated Form, TH-MeQ, Giving 2.9 wt·% Hydrogen Capacity

## Supplementary Material



## Notes

Table on the hydrogenation of MeQ in mixing experiments with 1-methylnaphthalene, NEC, DBT, and Q, figures on the characterization of hydrogenation products (GC chromatograms and mass spectra), ^1^H NMR spectra of DMeN and its hydrogenation product, and assignment of shifts in ^1^H NMR spectra corresponding to Figure 2 (PDF)

## Abbreviations

DH: TH decahydro and tetrahydro
DMeN: 2,6-dimethyl-1,8-naphthyridine
DMSO: dimethyl sulfoxide
FID: flame ionization detector
GC: gas chromatography
iPrOH: i-propanol
ISTD: internal standard
LOHC: liquid organic hydrogen carrier
MeQ: quinaldine
MS: mass spectrometer
NEC: *N*-ethylcarbazole
nH: *n*-hexane
NMR: nuclear magnetic resonance
PS: PV pore volume and size
Q: quinoline
SSA: specific surface area.

## References

[1] Aakko-Saksa P. T., Cook C., Kiviaho J., Repo T. (2018). Liquid Organic Hydrogen
Carriers for Transportation and Storing of Renewable Energy –
Review and Discussion. J. Power Sources.

[2] Díaz E., Rapado-Gallego P., Ordóñez S. (2023). Systematic Evaluation
of Physicochemical Properties for the Selection of Alternative Liquid
Organic Hydrogen Carriers. J. Energy Storage.

[3] Brückner N., Obesser K., Bösmann A., Teichmann D., Arlt W., Dungs J., Wasserscheid P. (2014). Evaluation
of Industrially Applied Heat-Transfer Fluids as Liquid Organic Hydrogen
Carrier Systems. ChemSusChem.

[4] Strauch D., Weiner P., Sarma B. B., Körner A., Herzinger E., Wolf P., Zimina A., Hutzler A., Doronkin D. E., Grunwaldt J.-D., Wasserscheid P., Wolf M. (2024). Bimetallic Platinum Rhenium Catalyst for Efficient Low Temperature
Dehydrogenation of Perhydro Benzyltoluene. Catal.
Sci. Technol..

[5] Kuutti K., Ghosalya M. K., Porri P., De Bellis J., Jokimies P., Singh H., Wang S., King G., Fernández-Catalá J., Schüth F., Ainassaari K., Huuhtanen M., Huttula M., Urpelainen S., Rautiainen S. (2025). Mechanochemical
Synthesis of Pt/TiO2 for Enhanced Stability
in Dehydrogenation of Methylcyclohexane. Catal.
Sci. Technol..

[6] Taube M., Rippin D. W. T., Cresswell D. L., Knecht W. (1983). A System of h y d r
o g e n - p o w e r e d Vehicles with Liquid Organic Hydrides. J. Hydrogen Energy.

[7] Jeong K., Yook H., Lee S. H., Han H. J., Jung Y., Han S., Shin S. Y., Choi M., Kwon S., Lee J. H., Kim S. J., Kim S. M., Han J. W., Park J. H. (2024). Benzyl-Methylbenzyl-Benzene:
Improving Hydrogen Storage and Release Performance of Dibenzyltoluene
Based Liquid Organic Hydrogen Carrier. Chem.
Eng. J..

[8] Crabtree R. H. (2008). Hydrogen
Storage in Liquid Organic Heterocycles. Energy
Environ. Sci..

[9] Amende M., Schernich S., Sobota M., Nikiforidis I., Hieringer W., Assenbaum D., Gleichweit C., Drescher H., Papp C., Steinrück H., Görling A. (2013). Dehydrogenation Mechanism
of Liquid Organic
Hydrogen Carriers: Dodecahydro-N-Ethylcarbazole on Pd(111). Chem.--A Eur. J..

[10] Gleichweit C., Amende M., Bauer U., Schernich S., Höfert O., Lorenz M. P. A., Zhao W., Müller M., Koch M., Bachmann P., Wasserscheid P., Libuda J., Steinrück H., Papp C. (2014). Alkyl Chain Length-Dependent
Surface Reaction of Dodecahydro-N-Alkylcarbazoles on Pt Model Catalysts
Alkyl Chain Length-Dependent Surface Reaction of Dodecahydro-N-Alkylcarbazoles
on Pt Model. J. Chem. Phys..

[11] Teichmann D., Arlt W., Wasserscheid P., Freymann R. A. (2011). Future Energy Supply
Based on Liquid Organic Hydrogen Carriers (LOHC). Energy Environ. Sci..

[12] Teichmann D., Arlt W., Wasserscheid P. (2012). Liquid Organic Hydrogen Carriers
as an Efficient Vector for the Transport and Storage of Renewable
Energy. Int. J. Hydrogen Energy.

[13] Teichmann D., Stark K., Müller K., Zöttl G., Wasserscheid P., Arlt W. (2012). Energy Storage in Residential
and
Commercial Buildings via Liquid Organic Hydrogen Carriers (LOHC). Energy Environ. Sci..

[14] Yang M., Dong Y., Cheng H. (2014). Hydrogenation Kinetics of N-Ethylcarbaozle
as a Heteroaromatic Liquid Organic Hydrogen Carrier. Adv. Mater. Res..

[15] Morawa E. K., Rentsch D., Friedrichs O., Remhof A., Zuettel A., Ramirez-cuesta A. J., Chi T. S. (2010). Hydrogenation of 9-Ethylcarbazole
as a Prototype of a Liquid Hydrogen Carrier. Int. J. Hydrogen Energy.

[16] Eblagon K. M., Tam K., Yu K. M. K., Tsang S. C. E. (2012). Comparative Study of Catalytic Hydrogenation
of 9-Ethylcarbazole for Hydrogen Storage over Noble Metal Surfaces. J. Phys. Chem. C.

[17] Lim S., Song Y., Jeong K., Park J. H., Na K. (2022). Enhanced Dehydrogenative
H2Release from N-Containing Amphicyclic LOHC Boosted by Pd-Supported
Nanosheet MFI Zeolites Having Strong Acidity and Large Mesoporosity. ACS Sustain. Chem. Eng..

[18] Gong X., Li L., Shi R., Zhang R., Jiang Z., Fang T. (2023). Novel Liquid
Organic Hydrogen Carriers with High Hydrogen Performance: NPhCZ/18H-NPhCZ. ACS Sustain. Chem. Eng..

[19] Zhang Y., Markiewicz M., Filser J., Stolte S. (2018). Toxicity of
a Quinaldine-Based
Liquid Organic Hydrogen Carrier (LOHC) System toward Soil Organisms
Arthrobacter Globiformis and Folsomia Candida. Environ. Sci. Technol..

[20] Wei Z., Shao F., Wang J. (2019). Recent Advances in Heterogeneous
Catalytic Hydrogenation and Dehydrogenation of N-Heterocycles. Chin. J. Catal..

[21] Ding X., Chen Y., Nan J., Dai H., Wang Y., Bai G., Qiu W. (2022). Ultrasmall Palladium
Nanoparticles Anchored on N-Doped
Nestlike Carbon Nanosheets for Selective Hydrogenation of Quinolines. ACS Sustain. Chem. Eng..

[22] Fujita K. I., Kitatsuji C., Furukawa S., Yamaguchi R. (2004). Regio- and
Chemoselective Transfer Hydrogenation of Quinolines Catalyzed by a
Cp*Ir Complex. Tetrahedron Lett..

[23] Zhou H., Li Z., Wang Z., Wang T., Xu L., He Y., Fan Q. H., Pan J., Gu L., Chan A. S. C. (2008). Hydrogenation
of Quinolines Using a Recyclable Phosphine-Free Chiral Cationic Ruthenium
Catalyst: Enhancement of Catalyst Stability and Selectivity in an
Ionic Liquid. Angew. Chemie - Int. Ed..

[24] Kim S., Lee C. G., Kim Y. T., Kim K. H., Lee J. (2020). Effect of
Pt Catalyst on the Condensable Hydrocarbon Content Generated via Food
Waste Pyrolysis. Chemosphere.

[25] Köhler K., Heidenreich R. G., Krauter J. G. E., Pietsch J. (2002). Highly Active Palladium/Activated
Carbon Catalysts for Heck Reactions: Correlation of Activity, Catalyst
Properties, and Pd Leaching. Chem. - A Eur.
J..

[26] Fan G., Wu J. (2013). Mild Hydrogenation
of Quinoline to Decahydroquinoline over Rhodium
Nanoparticles Entrapped in Aluminum Oxy-Hydroxide. CATCOM.

[27] Park I. S., Kwon M. S., Kang K. Y., Lee J. S., Park J. (2007). Rhodium and
Iridium Nanoparticles Entrapped in Aluminum Oxyhydroxide Nanofibers:
Catalysts for Hydrogenations of Arenes and Ketones at Room Temperature
with Hydrogen Balloon. Adv. Synth. Catal..

[28] Garbarino G., Bellotti D., Finocchio E., Magistri L., Busca G. (2016). Methanation
of Carbon Dioxide on Ru/Al2O3: Catalytic Activity and Infrared Study. Catal. Today.

[29] Campanati M., Vaccari a., Piccolo O. (2002). Mild Hydrogenation
of Quinoline1.
Role of Reaction Parameters. J. Mol. Catal.
A Chem..

[30] Fache F. (2004). Solvent Dependent
Regioselective Hydrogenation of Substituted Quinolines. Synlett.

[31] Campanati M., Casagrande M., Fagiolino I., Lenarda M., Storaro L., Battagliarin M., Vaccari A. (2002). Mild Hydrogenation of Quinoline 2. J. Mol. Catal. A: Chem..

[32] Sun Y., Fu H., Zhang D., Li R., Chen H., Li X. (2010). Complete Hydrogenation
of Quinoline over Hydroxyapatite Supported Ruthenium Catalyst. CATCOM.

[33] Sun B., Khan F.-A., Vallat A., Süss-Fink G. (2013). NanoRu@hectorite:
A Heterogeneous Catalyst with Switchable Selectivity for the Hydrogenation
of Quinoline. Appl. Catal. A Gen..

[34] Zhu D., Jiang H., Zhang L., Zheng X., Fu H., Yuan M., Chen H., Li R. (2014). Aqueous Phase Hydrogenation
of Quinoline to Decahydroquinoline Catalyzed by Ruthenium Nanoparticles
Supported on Glucose-Derived Carbon Spheres. ChemCatChem.

[35] Rahi R., Fang M., Ahmed A., Sánchez-Delgado R. A. (2012). Hydrogenation
of Quinolines, Alkenes, and Biodiesel by Palladium Nanoparticles Supported
on Magnesium Oxide. Dalt. Trans..

[36] Karakulina A., Gopakumar A., Fei Z., Dyson P. J. (2018). Chemoselective Reduction
of Heteroarenes with a Reduced Graphene Oxide Supported Rhodium Nanoparticle
Catalyst. Catal. Sci. Technol..

[37] Bianchini, C. ; Meli, A. ; Vizza, F. Hydrogenation of Arenes and Heteroatoms, In: de Vries, J. G. , Elsevier, C. J. , Eds.; Handbook of Homogeneous Hydrogenation, Vol. 2.; Wiley VCH: Weinheim, Germany, 2007.

[38] Sánchez-Delgado R. A., Machalaba N., Ng-a-qui N. (2007). Hydrogenation of Quinoline by Ruthenium
Nanoparticles Immobilized on Poly­(4-Vinylpyridine). Catal. Commun..

[39] Krajczewski J., Ambroziak R., Kudelski A. (2022). Formation and Selected
Catalytic
Properties of Ruthenium, Rhodium, Osmium and Iridium Nanoparticles. RSC Adv..

[40] Sand M., Skeie R. B., Sandstad M., Krishnan S., Myhre G., Bryant H., Derwent R., Hauglustaine D., Paulot F., Prather M., Stevenson D. (2023). A Multi-Model
Assessment of the Global Warming Potential of Hydrogen. Commun. Earth Environ..

[41] Graca I., Woodward R. T., Kennema M., Rinaldi R. (2018). Formation and Fate
of Carboxylic Acids in the Lignin-First Biore Fi Ning of Lignocellulose
via H - Transfer Catalyzed by Raney Ni. Sustain.
Chem. Eng..

[42] Mehranfar A., Izadyar M., Esmaeili A. A. (2015). ScienceDirect
Hydrogen Storage by
N-Ethylcarbazol as a New Liquid Organic Hydrogen Carrier: A DFT Study
on the Mechanism. Int. J. Hydrogen Energy.

[43] Sotoodeh F., Zhao L., Smith K. J. (2009). Kinetics
of H_2_ Recovery
from Dodecahydro-N-Ethylcarbazole over a Supported Pd Catalyst. Appl. Catal. A: Gen..

[44] Konda S. K., Chen A. (2016). Palladium Based Nanomaterials
for Enhanced Hydrogen Spillover and
Storage. Mater. Today.

[45] Pawelec B., Mariscal R., Navarro R. M., Van Bokhorst S., Rojas S., Fierro J. L. G. (2002). Hydrogenation
of Aromatics over Supported
Pt-Pd Catalysts. Appl. Catal. A Gen..

[46] Kustov L. M., Tarasov A. L., Kirichenko O. A. (2017). Microwave-Activated
Dehydrogenation
of Perhydro-N-Ethylcarbazol over Bimetallic Pd-M/TiO2 Catalysts as
the Second Stage of Hydrogen Storage in Liquid Substrates. Int. J. Hydrogen Energy.

[47] Ge D., Hu L., Wang J., Li X., Qi F., Lu J., Cao X., Gu H. (2013). Reversible
Hydrogenation-Oxidative Dehydrogenation
of Quinolines over a Highly Active Pt Nanowire Catalyst under Mild
Conditions. ChemCatChem.

[48] He T., Pei Q., Chen P. (2015). Liquid Organic Hydrogen Carriers. J. Energy Chem..

[49] Sánchez A., Fang M., Ahmed A., Sánchez-Delgado R. A. (2014). Hydrogenation
of Arenes, N-Heteroaromatic Compounds, and Alkenes Catalyzed by Rhodium
Nanoparticles Supported on Magnesium Oxide. Appl. Catal. A Gen..

[50] Mao H., Chen C., Liao X., Shi B. (2011). Journal of Molecular
Catalysis A: Chemical Catalytic Hydrogenation of Quinoline over Recyclable
Palladium Nanoparticles Supported on Tannin Grafted Collagen Fibers. J. Mol. Catal. A, Chem..

[51] Nie J., Zhu Z., Liao Y., Xiao X., Mauriello F., Zhang Z. (2022). Highly Efficient and Selective Hydrogenation of Quinolines at Room
Temperature over Ru@NC-500 Catalyst. Mol. Catal..

[52] Tan Y., Han M., Peng P., Sun Z., Shi J., Huang Y., Chen J., Bai L., Yang J., Chen Q. (2023). Strengthening
Spillover Hydrogenation of Quinoline Compounds over Platinum-Encapsulated
Amorphized HA Zeolite Catalyst. Mol. Catal..

