# Supplementary material for: Solvent-Free Hydrogenation and Dehydrogenation of Quinoline and Quinaldine for the LOHC Concept
Source: ACS Omega. 2026 Feb 19;11(8):14073–81. doi: 10.1021/acsomega.6c00675 (PMC12961470; doi:10.1021/acsomega.6c00675)
Supplement: Supplementary file 1 [file ao6c00675_si_001.pdf]

Supporting Information

Solvent-free hydrogenation and dehydrogenation of quinoline and quinaldine for the LOHC concept

Päivi Aakko-Saksa,*[a][b] Aino M. Mykkänen[a] and Timo Repo*[a]

[a] University of Helsinki, A. I. Virtasen aukio 1, PO Box 55, 00014 Helsinki (Finland)

[b] VTT Technical Research Centre of Finland Ltd, PO Box 1000, 02044 VTT (Finland)

Table S1. Hydrogenation of MeQ in mixing experiments with 1-methylnaphthalene (MeN), NEC, DBT and Q. Amount of catalyst 0.25 mol-%. H4 is tetrahydro and H12 is decahydro form of MeQ.

Catalyst	Substrates A+B	Pressure (bar)	Temperature (°C)	Time (h)	A: H4/H12 (%)	B Hydrog. (%)
Pt/C	MeQ+DBT	20	150	2	100 / 0	0
Pt/C	MeQ+1MN	40;30;20	150	2	100 (all) / 0	0
Pt/C	MeQ+Q	20	150	2	100 / 0	100 (H4)
Ru/Al_2O_3	MeQ+DBT	40;20	150	2	76;33 / 0	0
Ru/Al_2O_3	MeQ+1MN	20	150	2	25 / 0	0
Ru/Al_2O_3	MeQ+NEC	30	130	2	3 / 0	0

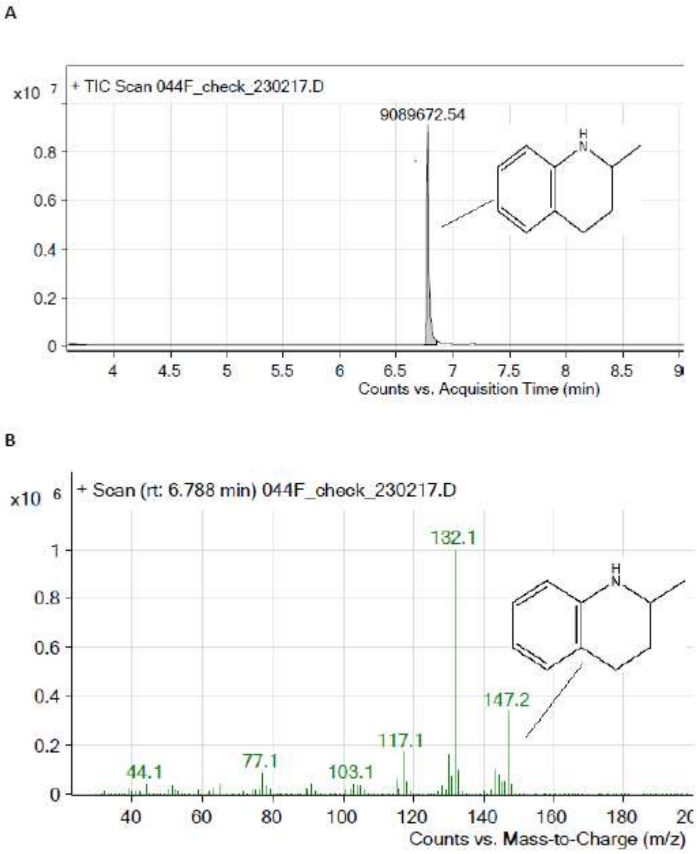

Figure S1. Product of hydrogenation reaction (entry 044) a) GC chromatogram b) mass spectrum showing peak mass, m/z of 147.1 for THQ.. This product was used in dehydrogenation reaction.

A

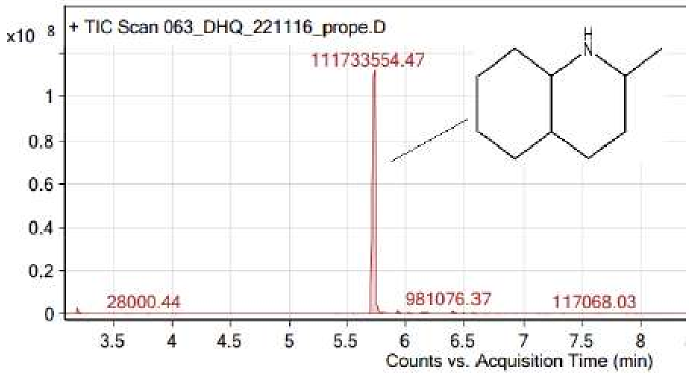

B

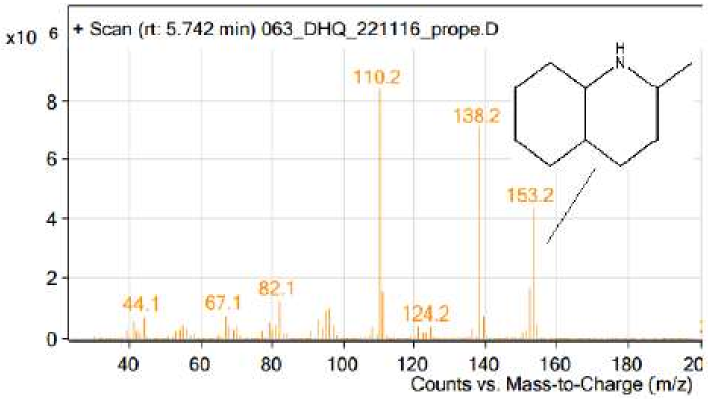

Figure S2. Product of hydrogenation reaction (entry 063) characterized a) GC chromatogram b) mass spectrum showing peak mass, m/z of 153.2 for DHQ.

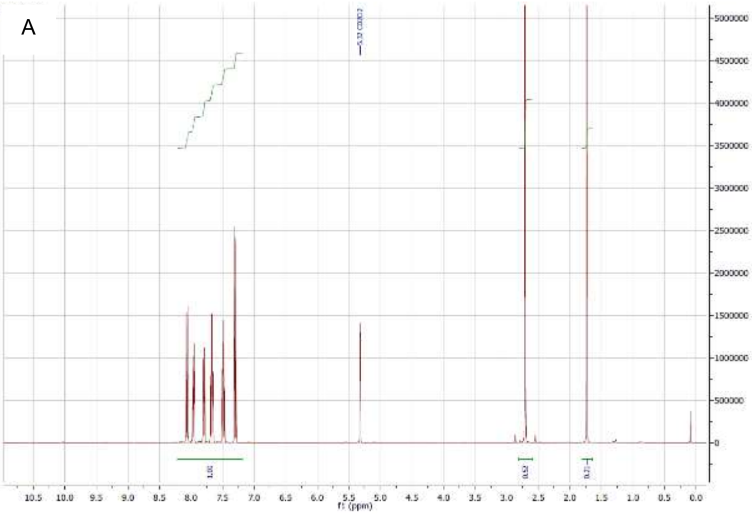

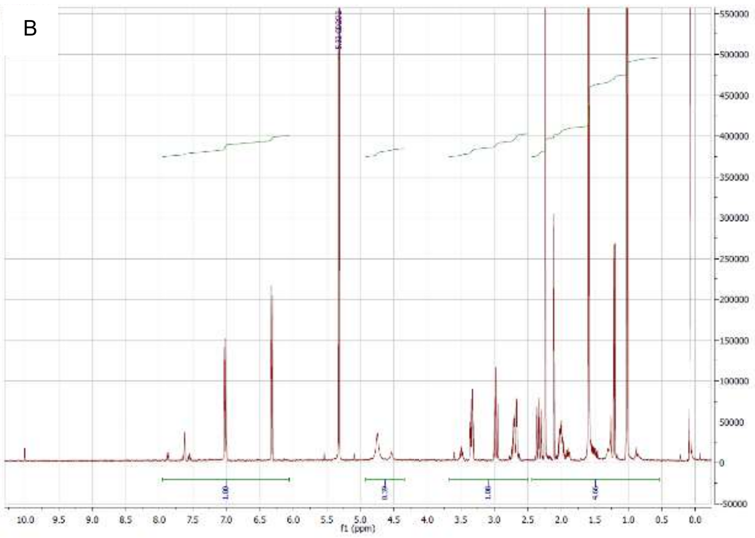

Figure S3. ^{1}H-NMR spectra of DMeN hydrogenation a) DMeN, integral of aromatic region 57.8% b) hydrogenation product, integral of aromatic region 14%.

Table S2. Assignment of shifts in ^{1}H-NMR spectra (in DMSO) for hydrogenated TH-MeQ product (py-TH-MeQ) and quinaldine (MeQ) corresponding to Figure 2 in the manuscript.

**Quinaldine (MeQ)**	**1,2,3,4-tetrahydroquinaldine (py-TH-MeQ)**
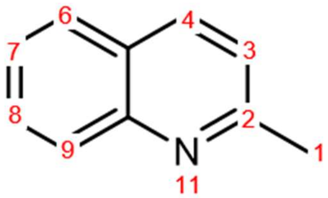	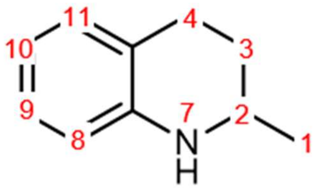

Quinaldine (MeQ):

Assign	Shift (ppm)
4, 9	8.3
6	7.9
8	7.7
7	7.6
3	7.4
1	2.7

1,2,3,4-tetrahydroquinaldine (py-TH-MeQ):

Assign	Shift (ppm)
9	6.9
11	6.9
10	6.5
8	5.5
7	4.1
2	3.3
4	2.5, 2.8
3	1.4, 1.8
1	1.2
